# Statutory Interpretation and Corpus Evidence: Tensions Between Linguistic Reality and Legal Meaning

**DOI:** 10.1007/s11196-025-10336-2

**Published:** 2025-08-02

**Authors:** Dana Roemling

**Affiliations:** Department of Linguistics and Communication, University of Birmingham, Edgbaston, Birmingham, B15 2TT UK

**Keywords:** Language and law, Legal linguistics, Legal corpus linguistics, Statutory interpretation, Ordinary meaning

## Abstract

This paper examines how linguistic meaning interacts with legal interpretation for the statutory term *child*, focusing on the 2013 Alabama Supreme Court case *Ex parte Ankrom*. In that case, the court held that *child* includes unborn individuals under chemical endangerment laws, a decision that raises important questions about how ordinary meaning is established. Using a triangulated, corpus-based framework grounded in linguistic theory, this study analyzes the term *child* across three corpora representing national, regional, and informal U.S. English. The methodology integrates semantic coding, grammatical context analysis, and lexical alternation to assess whether unborn individuals fall within the prototypical meaning of *child* in ordinary usage. The findings reveal a strong preference for interpreting *child* as referring to born individuals, with linguistic evidence supporting its extension to fetuses only in marked contexts. This tension between the court’s interpretation and the linguistic evidence highlights the challenges of aligning legal definitions with everyday language use. In emphasizing the role of empirical linguistic data, the study contributes to ongoing debates in legal corpus linguistics around frequency, representativeness, and contextual meaning. Ultimately, this analysis offers insight into how corpus linguistics can support statutory interpretation and reduce interpretive ambiguity without overstepping the boundaries between linguistic and legal expertise.

## Introduction

The purpose of this special issue is to examine the tensions between semiotic representation and legal interpretation across linguistic, cultural, and social domains. This study contributes to that aim by analyzing the interpretation of a statutory term in a contemporary U.S. American appellate decision, focusing on how linguistic meaning contrasts with legal application. Specifically, this study investigates the usage of the term *child* in relation to the Alabama Supreme Court’s (ALSC) decision in *Ex parte Ankrom*, which held that the term *child* extends to born and unborn individuals under chemical endangerment laws [1]. Through a corpus-based analysis, this study examines the ordinary usage of *child* across three corpora, offering insight into national, regional, and informal varieties of American English, to assess how the term is typically understood in everyday language. The findings reveal a gap between the court’s broad interpretation and the common linguistic understanding, highlighting the challenges of applying legal definitions that may not align with societal norms or language use. By emphasizing the role of empirical linguistic evidence in legal interpretation, this study contributes to ongoing debates about the interaction and tensions between law and linguistics, and the potential for corpus linguistics to inform more accurate and transparent legal reasoning.

## Language, Law and the Meaning of *Child*

Language and law are necessarily bound as natural language plays a critical role in the legal system, influencing everything from statutory interpretation to legal proceedings and interactions in court. As natural language is subject to ambiguity and vagueness and is reliant on context, so is law. One way that the legal system addresses these uncertainties is through statutory interpretation, the making sense of the law. This process is necessary because the language used in legal texts may not align with the real-world situations it is meant to regulate. Statutory interpretation is guided by rules of how meaning making is to take place [2]. As terms may have a specific meaning in law, or certain industries, that differ from everyday communication [3], meaning making is dependent on precedent and/or canons of interpretation [4]. Canons are a set of principles to direct legal interpretation and are an aid to applying the law [5]. One of these canons of construction is the textualist canon, which maintains that laws should be read in their ordinary meaning, based on the wording of the law [5].

One court case where the ordinary meaning of a word was relevant is *Ex parte Ankrom* [1]. In this case, the defendant, Ankrom, had tested positive for cocaine during her pregnancy, as did her son shortly after birth. Ankrom was subsequently found guilty of violating Alabama Code § 26-15-3.2, which punishes who “[k]nowingly, recklessly, or intentionally causes or permits a child to be exposed to, to ingest or inhale, or to have contact with a controlled substance […].” Ankrom did not deny the facts of the case but had appealed to the ALSC that the law had been applied incorrectly as it does not pertain to a fetus, but to a child. Ankrom argued that, given the rule of lenity, the law should have been construed narrowly. Ultimately, the ALSC upheld the ruling, arguing among other things that dictionary definitions of *child* included unborn children and that the interpretation was thus consistent with a plain-language interpretation [6]. Additionally, the court argued that the legislative intent of the statute was to guard children, both born and unborn [1]. This case thus exemplifies a core semiotic tension: while the court appeals to the apparent clarity of ordinary language, the language of the statute may be interpreted differently in actual linguistic practice.

There has been considerable criticism of the ALSC decision from various disciplines, including criminology [7], psychology [8] and law [9]. Researchers have argued that despite the ALSC upholding rulings penalizing drug use during pregnancy as child endangerment, the Supreme Court of the United States (SCOTUS) would have likely ruled differently [6], although notably these arguments were made before SCOTUS overturned the precedent set in *Roe v. Wade* [10]. Additionally, previous research has shown that the way legal professionals refer to a *child/fetus* impacts trial outcomes [11], thus further supporting the criticism raised against the ALSC decision.

Consequently, this case revolves around the question of what entity the word *child* conceptualizes in this statute. Given that the ALSC argued for a textual interpretation and citing plain language, it is crucial to establish the ordinary meaning of *child* in Ala. Code § 26. In particular, it is especially important to distinguish between two possible senses of the word *child*, born and unborn. In light of this background of the case, in this paper I investigate the following research question through a corpus-based analysis of the ordinary meaning of the term *child* in American English: Should the ordinary meaning of the word *child* in Ala. Code § 26 be interpreted as referring to children who are already born or to both children who are already born and who are unborn (i.e. fetuses)?

Further to answering the specific question related to the ordinary meaning of one term in one case, I also consider the more general question of how linguistic methodologies, especially corpus linguistics, can inform these types of debates around ordinary meaning. Before introducing the methods through which I address these questions, I therefore consider how ordinary meaning is traditionally established in a legal context and how linguistic methods have been applied and critiqued, especially corpus-based methods, and I also propose a new framework for the corpus-based analysis of ordinary meaning which addresses some of the limitations discussed in the background section. This framework also responds to the ongoing tension between linguistic representation and legal interpretation. I then apply the framework to the case at hand. In essence, I propose that to establish ordinary meaning it is important to consider how the term is used across multiple relevant varieties of language, represented by three distinct corpora, thus offering triangulation in determining the ordinary meaning of the disputed term.

## Ordinary Meaning: Linguistic-Legal Dissonance?

“Ordinary meaning” is a term of art referring to the principle, or canon, that, when words in a statute lack definition, they should be construed according to their natural meaning. This is also known as the ordinary-meaning rule and is based on the assumption that the word’s interpretation should reflect what people governed by the law would naturally understand [5]. The canon is a way of making sense of a law, but it also implies a decision is necessary in cases of divergent word meanings to understand which one is the ordinary meaning [12].

### Defining Ordinary Meaning

The ordinary meaning canon assumes “that words in legal texts should be interpreted in light of accepted and typical standards of communication” [13]. That, if words are undefined, we can rely on the average person’s understanding to be the ordinary meaning of the disputed term. However, while the canon itself and its intention may feel clearer in terms of applying a *natural word meaning*, there is no accepted definition of what ordinary meaning itself is [14]. This lack of definition highlights the tension between linguistic meaning and legal interpretation [see also 15] and the challenges that arise from it for interdisciplinary work.

Ordinary meaning is a legal concept, and it is meant to help understand legal language in the form of statutes. Consequently, it has been argued that establishing ordinary meaning should rest, for example, on the legislative context of what the legislator meant to regulate with a specific statute or on legal precedent [see 14]. This is part of the debate about whether ordinary meaning as a canon should be applied to construe a statute. If the decision has been set by precedent, there is no need to rely on the ordinary meaning canon to interpret the law. However, if indeed there is no sufficient definition of a word in a statute, the ordinary meaning canon is precisely intended to address this gap, ensuring that those governed by the law understand its scope, a democratic value praised for protecting the people it governs [16].

In an attempt to define ordinary meaning, SCOTUS describes it along the lines of “everyday parlance” [17], while Gries argues that ordinary meaning assumes a “contemporary ordinary reader” of a law that rests on the premise that the meaning is understood by readers now, whether or not that matches the meaning of a word at the time the statute was written [18]. This stands in contrast with Justice Scalia’s new textualism which relies on the meaning at the time of enactment [19]. And Solan relates that “ordinary meaning is by definition almost always going to be the most accessible one [i.e.,* meaning*], putting people on adequate notice of their rights and obligations, an advantage in its own right” [16]. But this has not always been the case.

The ordinary meaning canon emerged in 1878, gaining significant traction from the 1970s onward [20, 21]. Originally, SCOTUS referred to ordinary meaning as the sense of a word that people *could* understand, often allowing for a word to have multiple meanings [12]. Under this interpretation, if multiple constructions of a statute were possible, the rule of lenity was invoked, and the case decided in favor of the defendant as the ambiguity of the statute should not have the power to negatively impact the defendant [see 22]. Later, this default was lost, and more decisions were based on the most frequent meaning as the ordinary meaning [12]. Consequently, Solan & Gales propose two concepts of ordinary meaning [23]: One where the ordinary meaning is the word’s “most likely” conceptualization and the other where the speech community the word is used in would be comfortable in using the word in a certain conceptualization, the earlier version of ordinary meaning according to U.S. legal history. These two definitions accentuate the gap between a frequency-based definition, defining meaning by a word’s most frequent sense, versus a definition based on multiple potential word senses, where ambiguity is accepted as part of natural language and the subsequent task falls with the legislator to remedy the indecision [see 24]. However, there are more conceptualizations of what ordinary meaning is, for example, Lee and Mouritsen see five definitions of ordinary meaning in use by appellate court judges: A possible, a common, a most frequent, an exclusive, and a prototypical definition [see 3, 14]. So, consequently, if linguists wish to contribute empirical evidence to support the ordinary meaning determination, they must first agree on which meaning they are finding or assume to be the ‘right’ ordinary meaning. In light of the case at hand, it appears that the ALSC’s interpretation of the ordinary meaning of *child* includes a consideration of multiple possible senses, suggesting that the term can refer to both born and unborn individuals. However, the subsequent reasoning does not seem to fully account for the linguistic ambiguity. In this case study, I examine whether this interpretation aligns with linguistic facts.

### Establishing Ordinary Meaning

Assuming an agreement on which of the ordinary meanings one is looking to find, the act of determining ordinary meaning equally remains subject of ongoing discussion [12, 13]. There are three main ways to determine ordinary meaning linguistically, all of which rely on evidence external to the statute [25]. The first approach to decide which ordinary meaning a word takes is by employing linguistic intuition. The assumption is that “normal language users” naturally understand what a law means, which, in turn, allows the judge to simply make this call based on their linguistic intuition [13]. However, judges have been criticized for relying on intuitions that do not agree with how language is actually used [26, 27].

Alternatively, to resolve ordinary meaning disputes without relying on intuition, dictionaries can be used to determine the ordinary meaning of a term in question [13, 28, 29]. Dictionaries appear to be an objective way of finding the ordinary meaning by providing an external standard [21]. Although dictionaries often now rely on corpora for creation and selection and thus provide a partial insight into the actual usage of a word, they usually present word meaning without the context of natural language [28]. Furthermore, it would be easy for anyone wanting to guide the meaning making to find dictionaries that provide the relevant definitions for the favored legal decision [21] and use a meaning dispute to their advantage [11] by providing external ‘evidence’.

### Corpus Linguistics and Ordinary Meaning

The third approach to establishing ordinary meaning is Corpus Linguistics (CL). CL can be defined as a method for analyzing large, representative text collections, or corpora [30]. Legal CL, accordingly, refers to the use of such corpora to examine language in order to inform legal interpretation. Legal CL’s idea is the same as the use of dictionaries: to base the assessment of meaning on a more objective and more specific source [see 28]. By analyzing natural language data, legal CL offers a transparent and generalizable analysis, aiding empirical justifications [13, 21]. Such analyses were undertaken as early as 1993 to provide SCOTUS with linguistic evidence [31]. In *People v. Harris*, a case that pertained to the question whether “any information” includes false information or only true information, legal CL was used in the majority opinion to argue that “information”, without any modifying adjective, regularly encompasses both, true and false information, whereas the dissent used legal CL to argue that truthfulness is linked to the unmodified occurrence of the word [32, 33].

Consequently, legal CL has the potential to refine legal theory by supporting ordinary meaning making [27], but as with any tool it can be misapplied. This is why the suitability of legal CL in ordinary meaning cases remains debated [34–37]. Assessing legal CL’s appropriateness requires consideration of the case’s nature and whether word interpretation is in fact central to the case, which would allow for an application of legal CL [23]. In these cases, the legal decision still rests with the judge or jury of the case and legal CL should be considered an aid to empirical fact finding rather than the transfer of judicial power to a linguist.

However, there is a more detailed critique of how legal CL should be applied, which goes beyond the question of legal CL being applied in cases that are not actually about word meaning. Researchers have argued that analysis should consider the frequency of a particular sense in order to understand or uncover the ordinary meaning of a word in a statute [see, for example, 14]. The idea is that a word’s common meaning can be established by analyzing how frequently its meanings or senses are used and that the most frequent meaning is also the word’s common or ordinary meaning. Using legal CL as an empirical tool to evaluate the frequency of a meaning allows legal decision-making to be grounded in linguistic reality and provides a more objective alternative to intuition [3]. But, as Mouritsen contends, we should not blindly follow the most frequent meaning, but see what it can tell us about the intuition we may have had about a word’s use [38].

Others have argued that basing ordinary meaning making on frequency would obfuscate problems of linguistic analysis, as the frequency alone does not explain why a term is used [34], if it represents the relevant speech community [37] or that we assume the frequency absolves us from human judgment in the case [35]. While these criticisms are valuable, they do not imply that legal CL should not be applied or to not consider frequency of use, but that it needs to be applied as the tool it is: Allowing us to understand how people are using a word, which senses they are invoking, also commonly, and rather than assuming our intuition about language is correct, we can analyze both the frequencies in which a word meaning is used and its contexts. Klapper, for example, argues for the use of legal CL while saying that we can fall prey for the frequency fallacy [28]. He underscores that the corpus should be used in a way to understand the contexts of when senses are used by abstracting not from the statutory text to a case but from the case to the statute and seeing if ordinarily the concept of the case would be covered by the language in the statute [28]. A related argument is made by Slocum, who cautions against counting word meanings without considering the context [13]. Studies in legal CL have thus argued that word frequency must be evaluated in relation to the question at hand [23, 27].

Additionally, in terms of methodological criticism, it has been argued that legal CL looks empirical but is not objective enough [36]. This pertains, for example, to the sample size used to determine ordinary meaning. Previous studies have relied on samples ranging from 100 to 1000: Mouritsen sampled 500 and later 1,000 concordance lines [20, 38], Heilpern used 419 concordance lines (all results available) [3], while Lee & Mouritsen used 100 randomized concordance lines and later refer to 109 and 188 concordance lines [14, 27]. For these studies it has depended on context, and on time available, how many concordance lines were analyzed. Besides sample size, researchers have debated how to deal with infrequent word senses as the less frequent use of a sense may be due to factors like media prevalence of the frequent sense [34, 37]. Solan & Gales thus advocate for analyzing if people have other words at their disposal to instead refer to the infrequent sense [23]. Herenstein maintains that this approach brings in a certain bias, since the introduction of synonyms by the researcher is not objective and people may disagree with the synonyms [34]. However, it is a way to understand the usage patterns in the respective linguistic community and support the linguistic evidence.

Further criticism about legal CL’s application has been raised in terms of the representativeness of corpora [see 36]. Often, the Corpus of Contemporary American English (COCA) is used to determine the ordinary meaning in legal CL. Although it under-represents spoken and conversational data, it is a corpus with a variety of registers that is often taken as a mirror of general American English [39]. But Bernstein argues that generalized corpora, such as COCA, are inadequate for understanding the meaning of a term in law or in a more narrowly defined linguistic variety [37]. This ties in with discussions about authorship and audience of laws and whether corpora could and should match these conditions [27]. However, it has been argued that since meaning making is based on natural or ordinary meaning, the corpora should reflect this linguistic variety and be based on ‘ordinary’ language use rather than finding legal resources for ordinary meaning making [21]. If the basis of the ordinary meaning canon is the assumption that people governed by the law are meant to understand it, legal resources are counter-intuitive to finding this ordinary meaning, but a generalized corpus may offer these insights.

Goldfarb argues in an Amicus brief to the Utah Supreme Court that the corpus used for analysis in a legal case must represent the demographic group in question [40]. Additionally, as the law is a written text, it has been argued that ordinary meaning searches should rely equally on public, written text [14]. As CL bases its generalizations about linguistic varieties on the analysis of representative samples of natural language [41], the corpus selection needs to be in line with the case in question. Further, Phillips & Egbert argue that any generalization from the corpus data may only be done to the extent that it represents the specific speech communities in question [42].

Alongside corpus selection and methodological questions, researchers have explored the role of context for statutory interpretation. Context here can mean the linguistic context of a word (lexical, grammatical), its communicative context, but also the legal context of the law and the case [23]. Only the first two are contexts that CL can help analyze, the third is a question for jurisprudence. In terms of the linguistic side, Zoldan has posited that legal CL provides decontextualized results [36]. However, studies show that legal CL is very adept at taking context into account. For example, Goldfarb used an analysis of grammatical patterns for cases of disputed meaning to not only consider the meanings a word denotes, but also the contexts in which those meanings occur [43].

To conclude, for legal CL to be able to aid in cases of ordinary meaning making in a legal context, issues with methodology, representativeness of the chosen corpus, and context need to be addressed. This discussion also contributes to the broader themes explored in the journal’s special issue on tensions in law and legal semiotics, particularly examining the discord between statutory interpretation and corpus linguistics. It follows that we need more focused analyses and case studies to gauge the usefulness of corpus approaches in statutory interpretation. Therefore, this paper extends the methodology used in previous case studies by using not just COCA, but two additional corpora compiled to represent the linguistic community of Alabama where the defendant of the case lived. This paper thus addresses the criticism directed toward legal CL regarding its representativeness and its use of quantitative measures in meaning making. The combination of approaches and corpora shows the benefits of legal CL, offers triangulation in determining the ordinary meaning of *child* and addresses issues raised with previous applications of CL in language and law.

## Methodology

In order to investigate whether the ordinary meaning of the word *child* in Ala. Code § 26 is referring to born children or to both born and unborn children, the methodology for this paper is based on two different aspects of triangulation. One aspect is a three-step analysis framework to research the ordinary meaning of the word *child*. This is to address methodological criticism that assumes that corpus analyses purely relying on frequency and neglecting context will produce decontextualized results and that the infrequent use of a term cannot simply be taken as showing that a sense is not ordinary. The other aspect is considering the representativeness of corpora, which is another point of criticism in legal CL discussion. Consequently, this case study bases the analysis on three different corpora, two of which compiled for this study, thus ensuring that the represented linguistic varieties match the case at hand.

### Corpora

Given the issues raised about representativeness of corpora in previous research, this analysis is based on COCA, a Corpus of Alabama Newspaper Texts (CANT) and a collection from the short messaging service formerly called Twitter (Corpus of Alabama Tweets, CAT). COCA is a 1.1 billion word, freely accessible corpus collected from various sources, including newspapers and TV programs, to represent American English [39]. While it under-represents spoken and conversational sources, it does contain a variety of registers that help generalize across linguistic contexts. CANT has been collected via different newspapers from Alabama: The Anniston Star, the Cullman Times, the Decatur Daily and the News-Courier (Athens). All these newspapers were searched using the term *child* and any article containing *child* was downloaded. In the timeframe 2008 to 2021 this amounts to 8362 articles. CANT thus represents contemporary Alabama newspaper writing. CAT was collected using R and RStudio [44], scraping recent postings in a 120-mile radius around the geographic center of Alabama, which contain the word *child*. The postings are tweets, published on Twitter. 6857 tweets in English were collected on July 13th, 2021. All tweets were posted in July 2021 leading up to the day of collection. CAT represents Twitter postings from Alabama. CANT and CAT are collections of occurrences of the item in question, so their use is limited to this corpus analysis.

The rationale for using these corpora is as follows: any corpus analysis looking for the ordinary meaning should consider the language users in the case [40, 45]. In *Ex parte Ankrom* the relevant group of language users are the people of Alabama, as the law is governing their lives. The language of the defendant in the case is presumed to be American English, especially as spoken in Alabama. Hence, the three corpora provide insight into this group of language users. Should there be a considerable difference between American English and regional American English, this would become apparent through the use of CANT and CAT. CAT is used to additionally provide a more informal register to account for potential meaning differences between edited writing, such as newspapers, and personal communication. Still, the perceived audience of CAT is similarly public, as the tweets were published openly on Twitter. Since the case is from the 2010s, COCA, as well as CANT and CAT, provide insight into the relevant time period. Although COCA also has some spoken language data, most of its texts are written. CANT and CAT are similarly based on written data. Additionally, the rationale for drawing on these general-language corpora, rather than legal texts, is grounded in the aim of analyzing ordinary meaning. As legal interpretation in this case hinges on how *child* is used in everyday language, corpora reflecting general usage are best suited to capturing that meaning in context.

As the corpora in this analysis are composed of public data, this might pose an issue when researching taboo topics as these might not be adequately represented in public data. Although the topic of unborn children appears somewhat private, the question here is whether the usage of the word *child* in this public domain can aid in understanding its ordinary meaning or whether people would choose not to use the word *child*. This does not seem to be the case, so it is assumed that *child* does not fall under the scope of a taboo.

For COCA, sampling was done via CQPweb [46] given its availability to the researcher, while for CANT and CAT LancsBox [47] was used. 250 random concordance lines with the node *child* were used per corpus, totaling 750 lines. All lines included *child* as a noun, but not as a prenominal modifier, ensuring parallel use to the statute in question. Typos, names and duplicates were disregarded. Lines were also disregarded if *child* was used in the context of child endangerment, since this is the original case question so as not to skew results.

### Three-Step Framework

First, each concordance line was coded as either *born*, *unborn*, *neither* or *unclear*, which Phillips & Egbert call the “minimalist approach” [42], henceforth minimal coding approach (MCA). In this approach, node words are only coded for the main semantic categories of interest. This was done for all 750 instances of the node *child*, which is within the scopes of previous research in terms of sample size.

Secondly, grammatical and semantic patterns surrounding the word *child* were analyzed to see if certain senses are linked to certain patterns. Several grammatical patterns were coded during the analysis, as suggested by the Corpus Pattern Analysis (CPA) [48]. The approach helps to systematically define the patterns a word occurs in and link those patterns to different senses. The patterns analyzed include valency, voice, agentivity, pre- and post-modification of *child*, and determinatives. Coding was based on definitions by Huddleston & Pullum [49] and Hanks [48] and ensures any ordinary meaning developed from MCA can be evaluated in grammatical context.

Lastly, a “double dissociation”, so named by Solan & Gales, was explored as it is important to understand why a given sense may be infrequent [23]. Solan & Gales analyze if the infrequent usage of a particular sense is based on people using similar, but different, terms to refer to the required sense. This would show that people do have the need to talk about the sense in question but have a different linguistic preference for its conceptualization (e.g., using fetus to refer to unborn children). The present study analyzes whether people have alternative items available to refer to unborn individuals to explore whether any infrequent use of a specific sense of *child* is because people choose other conceptualizations. For this, 200 concordance lines with the nodes *baby*, *embryo*, *fetus* and *infant* (50 each) were analyzed using MCA, based on COCA. ​​The related terms in this analysis were derived by (a) noting any such terms during the analysis of *child* and (b) searching the Alabama code for terms referring to either young or unborn individuals by careful reading of relevant statutes.

## The Ordinary Meaning of *Child*

This section focuses on the application of the three-step framework. Initially, the word *child* is coded for the main semantic senses, born, unborn, neither or unclear, before a contextual analysis addresses grammatical context. The last step is evaluating whether people have alternative items at their disposal when referring to a particular sense of *child*. This analysis directly addresses the guiding research question of whether the ordinary meaning of *child* in Ala. Code § 26 refers solely to born children or also includes unborn children, thereby engaging with the broader tension between linguistic usage and legal interpretation.

### Minimal Coding

To analyze *child*’s ordinary meaning and understand whether *child* has preferred or default senses referring to individuals pre- and / or post-birth, each line was coded to reflect whether *child* refers to

1. A born individual,
2. An unborn individual,
3. Neither, or
4. A category for unclear cases,

following the minimalist coding approach. This semantic categorization was completed prior to the grammatical analysis. While it is difficult to separate grammatical context from semantic interpretation, the initial annotation focused on discourse-level meaning, aiming to assign each instance to one of the four categories based on its most salient, contextually-supported interpretation. The subsequent grammatical analysis was then used to explore whether particular senses are associated with recurring structural patterns, rather than to determine or validate the semantic categories. Several examples are given below to illustrate the distinctions.

A born individual can be conceptualized as having obvious agency, biological independence, doing things themselves that would require them to be born, being of a certain age, or existing in places and times outside the womb. An example^1^ from COCA is:

(1) “The day you brought me a picture of a woman and a child fleeing on a road lined with trees […]”.

An unborn individual can be conceptualized by reference to places or times within the womb or for instance by referring to pregnancy:

(2) “No surprise there, as rumor had it she was with child”.

Although being coded as unborn, some instances conceptualize a born individual, such as 3. Despite the sentence being written while the *child* is still unborn, it envisions a born individual for whom the parents wait, and who exists in the future. Still, these nodes were coded as unborn as at the time the *child* was still unborn.

(3) “Days later I found out he was engaged to another woman who was pregnant with his child”.

Cases in which the sense was ambiguous were marked as unclear:

(4) “A gentleman who has come forward, […], who has taken responsibility for the paternity of the child”.

In the fourth category instances of polysemous use of *child* were coded as “neither”, like ‘just ignore my message child.’

Turning to the results, of the 750 analyzed concordance lines, 668 reference a born child, 34 an unborn child, 36 are unclear and 12 denote neither sense. A summary is provided in Table 1. There it can be seen that 89.1% of concordance lines conceptualize the born sense and 4.5% the unborn sense. Although coding for the unborn sense included future conceptualizations as borderline cases, very few uses of *child* denote the unborn sense. Neither and unclear senses account for 6.4% of conceptualizations.

**Table 1:** Senses of *child* in count and percent per corpus

	COCA	CANT	CAT	Average Percentage Overall
Born	217 (86.8%)	232 (92.8%)	219 (87.6%)	89.1%
Unborn	17 (6.8%)	9 (3.6%)	8 (3.2%)	4.5%
Unclear	16 (6.4%)	8 (3.2%)	12 (4.8%)	4.8%
Neither	0	1 (0.4%)	11 (4.4%)	1.6%
Total	250	250	250	

Across the different corpora, there is a slight difference in the distribution of senses, however most of the instances of the word *child* in each corpus references a born individual. This slight variation can be seen in Table 1.

The informal CAT shows more use of the neither sense and COCA shows the most uses of both unclear and unborn senses. The regional corpora show somewhat less use of *child* in the unborn sense. COCA has the most use of the unborn sense, 6.8% of concordance lines, whereas CANT and CAT show 3.6% and 3.2% of the unborn sense respectively. COCA also has most instances of the unclear sense of the three corpora (6.4%), while CANT has 3.2% and CAT has 4.8%. CAT shows more polysemous use of *child*. Concluding, *child* is used most frequently in the born sense and only marginally in the other senses. Thus, *child* can refer to an unborn child, but in these corpora this is very rare and, in general, the dominant meaning of *child* is stable and clear cut denoting the born sense. This suggests a default born sense for the word *child*.

While this result shows a strong default for *child* referring to born individuals, this pattern may in part reflect the significantly longer duration of childhood post-birth compared to the relatively brief prenatal period. As such, the higher frequency of references to born children may be influenced not only by conceptual salience or legal relevance, but also by the temporal asymmetry between the two referential states. This limitation should be taken into account when interpreting frequency data as indicative of ordinary meaning, and it points to the need for a more nuanced, qualitative analysis of context and usage, which follows in the next section.

### Contextual Analysis

For the next step of the analysis, the grammatical patterns around the node word *child* were analyzed. In the statute *child* is in a direct object position, having a post-modifier infinitive construction and a determiner, but no modifier: who “[k]nowingly causes a child to be exposed to a controlled substance” [50]. Understanding this particular grammatical context may allow us to link senses to it.

This step was done by coding the grammatical context around the node word following CPA [43, 48]. For the example “Every child is different,” this would mean the grammatical context was coded as *child* being in subject position and that *child* is preceded by a determiner. Table 2 shows the summary of syntactic positions for each sense. Across all corpora, *child* is mostly used as a direct object, in all senses. Therefore, the grammatical position of the statute cannot be explicitly linked to a specific sense. The main difference between the unborn and the born sense is that *child* is used less in a subject position in the unborn sense. This could be due to semantic agency on part of the individual, which is semantically more likely to occur for a born individual.

**Table 2:** *Child* sense per syntactic position - all corpora

	Born Sense	Unborn Sense	Unclear	Neither
Subject	178	5	6	2
Indirect Object	10	0	2	0
Direct Object	217	14	19	6
Subject/Object Complement	56	1	0	1
Adjunct	207	14	9	3
Total	668	34	36	12

This analysis step further looked at the use of passive and *child* having the semantic role of the agent; however, both appear marginally. Additionally, the post-modification of *child* varies, and, in many instances, there is no post-modification. For the unborn sense there is no infinitive construction like “to be exposed to” as in Ala. Code § 26-15-3.2. There are 14 instances of this in the born sense (2.1%). There is one infinitive construction in the unclear sense (2.8%). This could mean the unborn sense would not take such a construction; however, since only little use of the construction is made overall, this does not seem indicative.

**Fig. 1:**
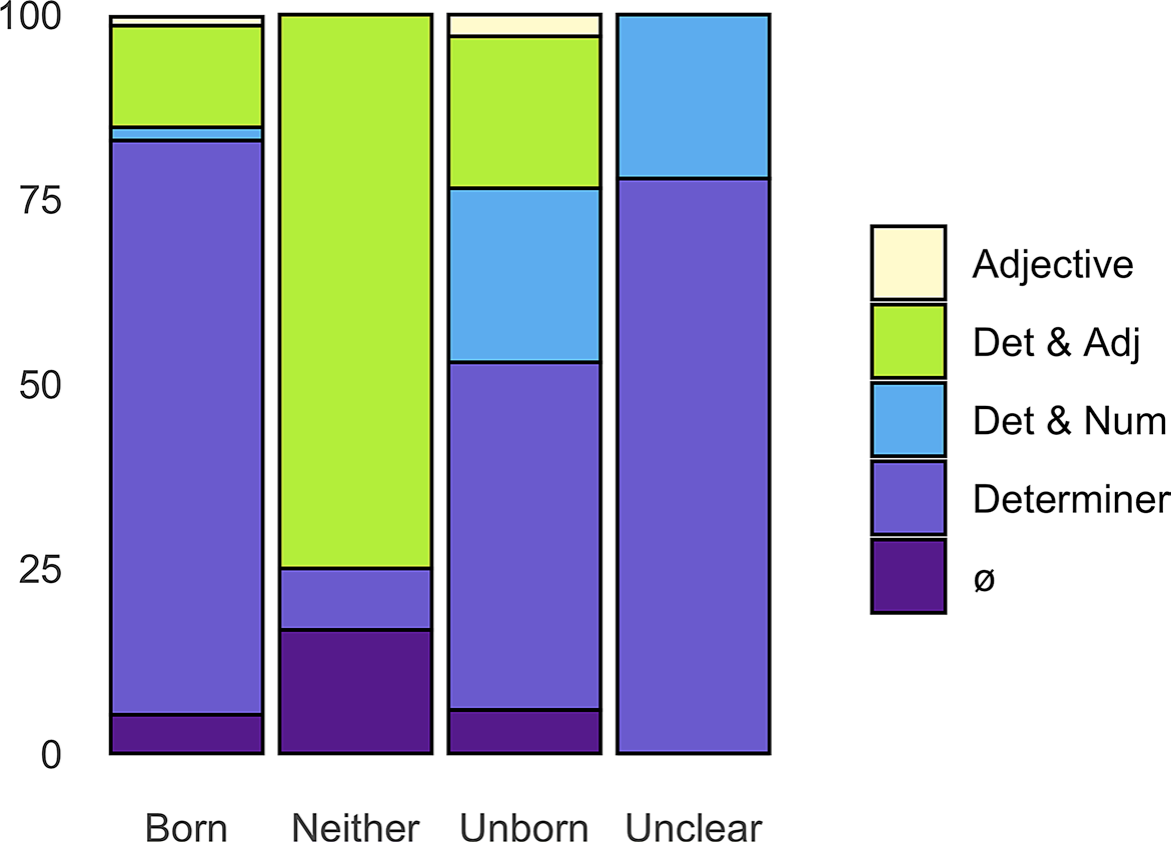
Premodification of *child* per sense in percent– all corpora

For the grammatical context, the context preceding *child* was analyzed further. The node word is preceded in 94.8% of concordance lines. Of these, the majority is preceded by a determiner, like “a child” or “her child” (75.1%). The unborn sense has lower determiner numbers, only being preceded by a determiner in 47.1%. The neither sense shows the highest number of ‘determiner + adjective + child’ constructions with 75%, where the other senses average on 13.4%. Figure 1 shows the overall usage in premodification and preceding tokens of *child* in all corpora and senses.

When numerals get used, constructions follow patterns like ‘expect + determiner + numeral + child’ or ‘pregnant with + determiner + numeral + child’. In the unborn sense, 23.5% of instances occur with ‘determiner + numeral’, in the unclear sense it is 22.2%. The neither sense does not occur with a numeral and for the born sense only 1.8% of nodes are accompanied by ‘determiner + numeral’. In comparison, the unborn and unclear senses show more ‘determiner + numeral + child’ constructions than the other senses. Overall, this shows there is variability in the patterns around *child* in all senses, however the unborn sense is more likely to co-occur with *child* not in subject position and to occur with some modification after the determiner such as an adjective or a numeral.

**Fig. 2:**
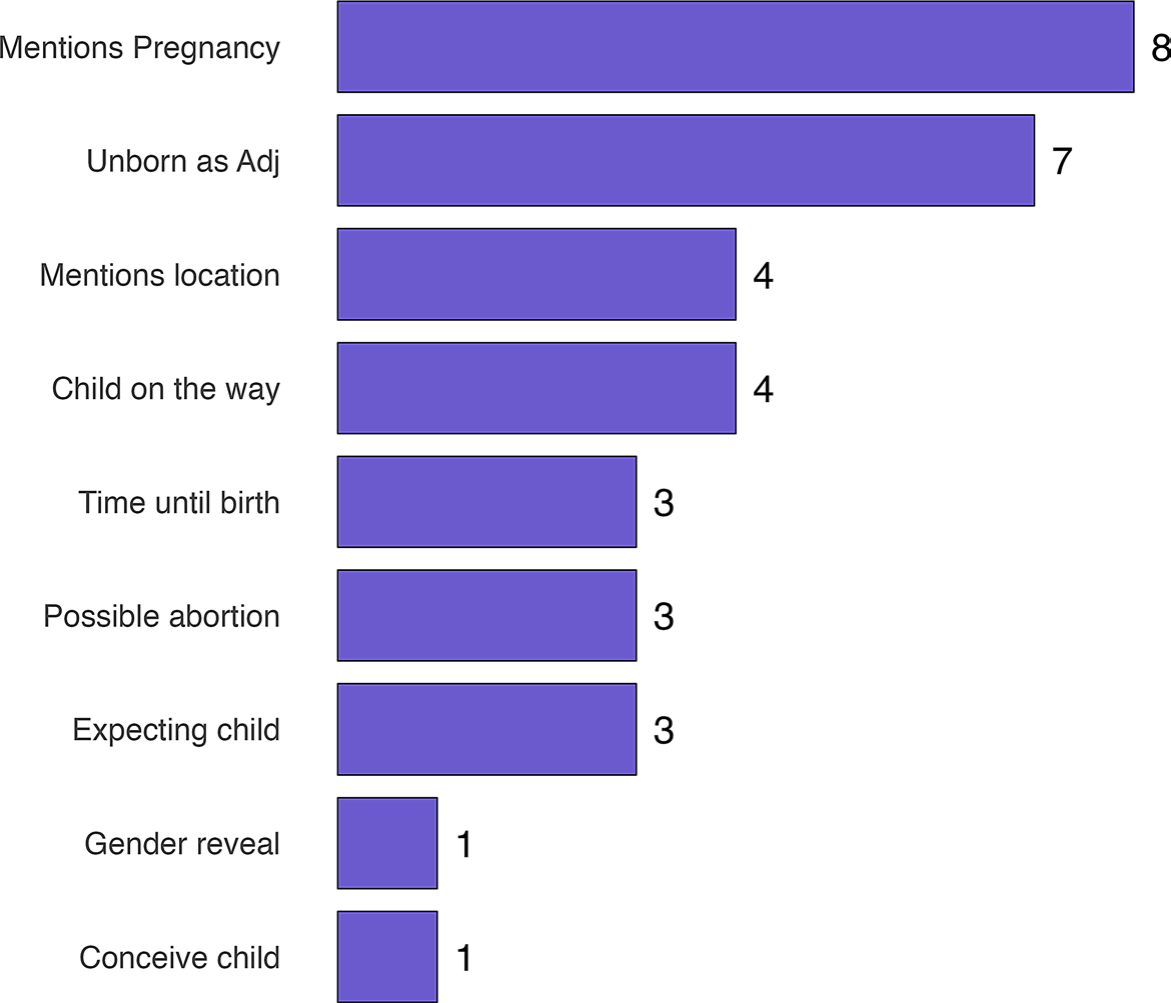
Overall modifications of *child*– unborn sense

Lastly, because of the marginal use of the unborn sense, the context of all unborn instances was analyzed. It was found that each instance of the unborn sense is used with a modification, either as a premodification to the word *child* or as a contextual modification in a pragmatic sense, and none come without modification. This relates to the linguistic notion of markedness, where the unmarked form is the common (or dominant) sense, whereas the marked form is the nontypical sense [51, 52]. This could pose a hierarchy on the senses of the word, from regular to marked. A premodification in this sense would be “unborn child” with an adjective that marks the sense, whereas a pragmatic modification can be seen in example 5 and Fig. 2 summarizes these findings. In example 5, child is an adjunct and modifies the phrase referring to the pregnancy:

(5) “That’s how I’ve started my day since I got pregnant with my first child”.

Of all modifications seven are premodifications in the form of an adjective, 26 are by context. Most modifications hence are indirect. Besides “unborn” the adjective “yet to-be-born” is used, which is grouped with the “unborn” adjectives. “Mentions location” in Fig. 2 designates a reference to the womb. “Child on the way” includes one instance of “is with child”. Crucially, there are no instances of the unborn sense having no modification. Thus, *child* in the unborn sense does not occur alone, it is accompanied by a modification that denotes the specific sense. Given that the statute uses *child* without a premodifying adjective and without a specific context, this statute provides the structure most used with the born sense.

### Double Dissociation

Finally, the last step of this analysis was to evaluate the infrequent use of the unborn sense. The question is whether it is just not within peoples’ realities to refer to unborn children more frequently than observed in the corpora or whether the infrequent usage may be due to the fact that people have other preferred linguistic items to use instead. For this, the following items were considered for analysis: baby, (human) fetus, infant, unborn child, unborn, incompetent and embryo. Incompetent denotes a different legal concept and was disregarded, as were unborn and unborn child as these have already been part of the analysis. That left *baby* and *infant* (derived from the corpus data) and *embryo* and *fetus* (Ala. Code § 26-23-3; § 26-17-706) for analysis.

Since CANT and CAT were collected searching for the node *child*, they did not contain adequate amounts of other conceptualizations. For this analysis, hence, COCA was used, and lines were coded with MCA as *born*, *unborn*, *unclear* or *neither*. *Child* was by far the most frequent of the analyzed words with 225 instances per million words, followed by *baby* with 108 instances per million words. *Embryo*, *fetus* and *infant* in comparison were rather infrequent and appeared in fewer documents within the corpus, which limits their dispersion [see 53]. It is unsurprising, however, that *embryo* and *fetus* are used more marginally, as they refer to an individual in a limited time span of their existence. *Child*, however, can be used up to 18 years (and more) and thus allows for many more contexts. The polysemous use of the words *child*, *baby* and *infant* also add to their usability.

**Fig. 3:**
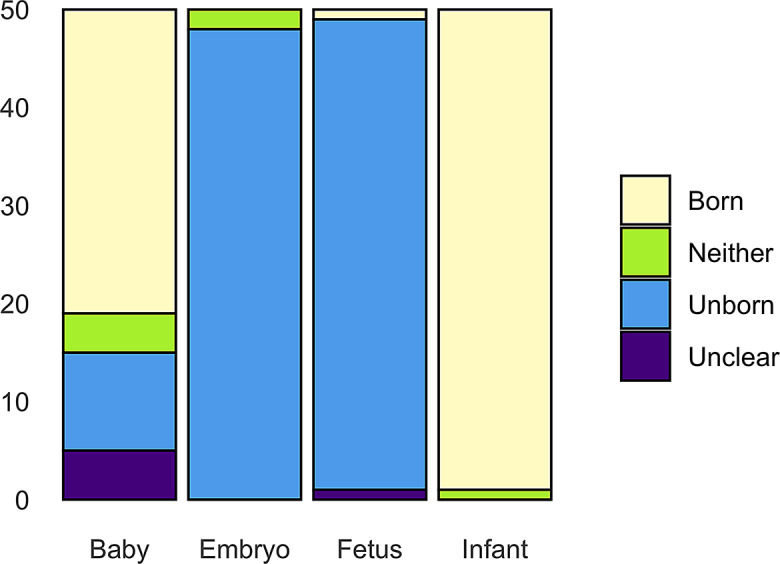
Alternative conceptualizations to *child* - all senses

For the double dissociation analysis, 50 lines per conceptualization were coded. For *embryo* and *fetus* non-human uses were discarded so that comparability to the statute was given. Additionally, just as with *child*, names, polysemous use or composite nominals were excluded. The following are examples of two of the senses and Fig. 3 shows the senses of alternative nodes.

Unborn:

(7) “[S]he was approaching [a shop] when the *fetus* inside her shifted from her left to her right side”.

Born:

(8) “At one child care site, she saw a 6-month old *baby* who looked at her with outstretched arms”.

In 96% of concordance lines, *embryo* and *fetus* refer to an unborn individual, whereas *infant* is in the born sense in 98% of cases. *Baby* is the only alternative that is not as clear-cut as 62% denote the born sense and 20% denote the unborn sense. Still, it is used primarily to conceptualize the born sense. *Baby* shows more variability in senses than *child* does, with the latter exhibiting 89.1% for the born and 4.5% for the unborn sense. *Fetus* also takes the premodifier of “unborn” to denote a specific conceptualization. In the context of *fetus*, viability is mentioned three times, conceptualizing a life outside the womb. Summarizing, this shows there are lexical resources at speakers’ disposal to refer to unborn individuals. Although the term *child* can span both prenatal and postnatal stages, the low frequency of unborn-specific usage suggests that speakers may conceptually distinguish the unborn and choose alternative expressions to reflect that distinction.

## Discussion and Conclusion

This paper has provided both an overview of the current debate on legal CL as well as an analysis of the ordinary meaning of the word *child* in *Ex parte Ankrom*, highlighting the ongoing tension between linguistic evidence and legal interpretation. The legal case judged that *child* includes unborn individuals in its scope for protection from chemical endangerment. While this may be the legislator’s intent, the ALSC argued that the ordinary meaning of *child* would include unborn individuals. However, analyzing the usage of *child* in three corpora shows that in most instances *child* refers to a born individual and only marginally, in specific contexts, to unborn individuals. Crucially, there are no instances of the unborn sense without modification and the born sense is the unmarked sense. This suggests that the default or ordinary interpretation of the word *child* is the born sense in the corpora employed here. People do use the unborn sense and find this usage acceptable, *if* there is contextual modification to designate this. This shows that an ordinary reader of the law is most likely to interpret *child* referencing born children. Even if one rejects the idea that ordinary meaning corresponds to a word’s most frequent or default sense, this analysis shows that the interpretation suggested by the ALSC - that *child* can also denote unborn individuals - fails to capture the complexity of actual language use where prototypically people understand *child* as born unless there is grammatical or contextual modification to evoke the unborn sense.

To explore ordinary meaning, this paper has used a minimal coding approach, a corpus pattern analysis, and a double dissociation analysis to understand the usage of *child* in its senses and different grammatical patterns. The analysis relied on three corpora, two regional and one informal, to triangulate how *child* is used in different linguistic varieties. The usage of *child* in these corpora would suggest the ‘ordinary’ person conceptualizes a born individual when reading the chemical endangerment statute. Laws should provide the reader of a statute with advance warning of what is allowed and what is forbidden [13], which, in this case, the statute may not have fully achieved. The linguistic evidence presented here raises important questions about whether the defendant’s claim of ambiguity was appropriately considered by the ALSC. While the interpretation of *child* as referring to both born and unborn individuals is linguistically plausible, given also the term’s ALSC acknowledged polysemy, the data does not clearly support that reading as the most typical. If the ordinary meaning canon had been applied historically, this could have prompted further consideration of ambiguity. The modern application of the ordinary meaning canon tends to prioritize the most frequent sense of a word, and in this case, the linguistic evidence suggests that the most common interpretation of *child* aligns with the born sense. These findings underscore that the relationship between linguistic evidence and legal interpretation could have benefited from a more nuanced consideration of the complexities of language use.

Consequently, this paper highlights that tension between legal interpretation and linguistic reality. While the court in *Ex parte Ankrom* appeals to the plain language of *child*, the corpus evidence suggests that usage is more variable, context-sensitive, and heavily skewed toward born individuals in ordinary language. The purpose of legal interpretation in this case was to extend the statute’s scope to include unborn individuals by invoking plain-language reasoning. However, the linguistic data complicate that reading, showing that *child* does not typically refer to the unborn in everyday usage. This disconnect between presumed meaning and actual usage highlights the need for empirical methods in legal interpretation and invites reflection on the normative vs. descriptive roles of language in law.

In *Ex parte Ankrom*, the court addressed not only a legal or potentially socio-political question, but also relied on dictionaries to inform its understanding of the ordinary meaning of the term *child*, ultimately deeming the rule of lenity inapplicable [1]. However, the court did not conduct an analysis of usage frequency or exclusivity in a linguistic sense; instead, it relied on dictionary definitions and legal precedent to assert a plain-language reading. This highlights the relevance of incorporating linguistic evidence into legal decisions. Although legal CL has been criticized in its application, the linguistic evidence learned through legal CL is an important argument for exploring legal CL further. Given the analysis Danet provided, we can see that the word choices in a case concerning the beginning of life can have an influence on trial outcomes [11], as it did in *Ex Parte Ankrom*. The linguistic analysis provided here also shows that whilst frequency has been called into question [35, 37], it remains a useful indicator of actual language usage, especially when considered within the appropriate contextual and semantic framework. Previous research has noted this as well [23, 27]: frequency is an important tool to understand language use when taking into consideration the context and potential double dissociation of terms.

Importantly, legal CL highlights the problems arising from a lack of definition of ordinary meaning [16], both within legal CL itself, but also in the legal community in general. Arguments can be made for specific senses of a word, proclaiming them the ordinary meaning of a term, without questioning whether there are other relevant meanings or whether it is the appropriate meaning for the case at hand. In *Ex parte Ankrom* the court argued that unborn individuals are part of the meaning of *child*, and this argument is not wrong [1]. Yet, it neglects the linguistic reality that the default concept evoked is a born child and, with that, fails to acknowledge ambiguity and a definitional problem when it comes to ordinary meaning.

Previous studies in legal CL have criticized the use of COCA, because it would not represent the relevant group of language users, genre, audience or medium [36, 37]. To address the potential problems with corpora selection, this study has used two additional corpora to investigate whether different corpora would provide different results. Toward that end two corpora have been compiled for the case analysis, specifically reflecting a narrower understanding of the group of language users. Although future research may well expand further on the issue of representativeness [see 54], this study demonstrates that COCA and regional English corpora show a very similar distribution of senses of the word *child*. For this case, there is no difference in usage in the general American variety and in regional varieties. Additionally, the corpus based on Twitter data provided a more informal, though still written, sample. But again, COCA and the informal CAT provided the same conclusions about the usage of *child*. This study has zoomed in on regional linguistic use and informal register in addition to the general American English variety present in COCA and has found that there is consistency across registers. This triangulation helps to understand that in this case there is a strong default for interpreting *child* as born and that, while multiple corpora representing different varieties were used, the conclusions are the same. Although COCA may have sufficed in this case, it demonstrates that the use of specialized corpora is a purposeful tool for strong results, which should be explored in further case studies.

This study focuses on ordinary-language corpora and does not include a systematic analysis of legal corpora. While this approach aims to illuminate how the term *child* is used and understood in general language, it does not capture the full range of legal meanings the term may carry within legal discourse. As legal interpretation is ultimately the domain of legal professionals and given that legal terms often acquire specialized meanings distinct from ordinary usage, a more comprehensive study could incorporate legal corpora or case law to identify how *child* is construed in legal contexts. Future research might explore this legal dimension more directly, for example by examining judicial opinions, statutory definitions, or doctrinal commentary that explicitly interpret the term *child*. Such work could help clarify the relationship between ordinary and legal meanings and shed further light on how courts navigate that distinction.

Additionally, this study focuses on the ordinary meaning of the term *child* as used in everyday language at the time of the legal decision, rather than tracking its legal evolution over time. While legal definitions have shifted significantly, such as in *Roe v. Wade* [10] and *Dobbs v. Jackson Women’s Health Organization* [55], this paper does not attempt to analyze those doctrinal developments. Future work might explore how changes in legal interpretation influence, or are influenced by, broader linguistic usage. Similarly, this paper analyzes ordinary language within the context of a state-level case in Alabama. It does not aim to reconcile divergent legal definitions across jurisdictions, such as federal and other state statutes. While such inconsistencies are highly relevant in legal interpretation, the focus here remains on linguistic patterns in general language use as they relate to ALSC’s ruling regarding plain language.

Furthermore, this case study has relied on different approaches to analyze the linguistic data to counter the problem of subjectivity and ‘half-empiricism’ [37]. Understanding the different senses of *child* by a minimal coding approach, linking the senses with grammatical patterns, and understanding the infrequent use of senses provides an overall picture of the conceptualizations that come with the word *child*. While the coding will always rely on the input and categorization of the coder [see 36], looking at the different senses regarding their grammatical patterns and alternative conceptualizations minimizes partiality and puts emphasis on the grammatical context of the word in question. Although in this case study the strongest results are the distributions of senses alone as there is not much variation, the combination of approaches shows that the grammatical pattern analysis can be a useful tool to relate corpus findings and statutory text.

Decontextualized results are what some critics have called the conclusions of legal CL, but this study has shown that the linguistic context of the statute and of the results can be linked. It provides an understanding of how an average reader, like the average authors in the corpora here, would use the word that has proven to be problematic in the statute. Regional linguistic varieties and usage on social media are strong evidence for a default interpretation of *child*. The combination of corpora and approaches, however, means the case study here is less accessible to legal professionals than a simple cross-check in COCA would be.

The results in this study show a default use of the word *child*. This outcome would be even stronger if constructions like ‘expecting + determiner + numeral + child’ had been coded as born, since they reference the time the child is actually with their parents or, as Danet details, readers consider ‘aliveness’ a given [11]. Further, this analysis did not look at the specific construction of ‘causing or permitting a child’ and hence found only marginal use of this construction. While other CL studies have narrowed their node context [e.g., 56], it is argued that in this case the issue is with the conceptualization of *child* before and after birth and not which verb was used surrounding it. Further analysis, though, could take this into account. Furthermore, note that most of the modification of the unborn sense is indirect and not by grammatical means. Additionally, the double dissociation analysis could have been expanded to regional and informal language use as well, which would have strengthened the results of this analysis step.

This corpus application is not intended to replace a judge, but to help base a legal decision on linguistic facts, so that meaning making is not based on intuition or dictionaries. Although legal CL has been criticized and its applicability doubted, this case study shows that legal CL is able to assist in finding the linguistic evidence needed to support the court when dealing with questions of ordinary meaning [16]. This highlights the ongoing tension between linguistic analysis and legal interpretation, suggesting that a more data-driven approach can complement traditional legal reasoning. Consequently, this case study has illustrated that a combination of corpora and approaches is particularly well suited to provide these solid results.

Summarizing, it can be said that the methodological contributions of this study are two-fold. First, the use of three corpora strengthens the results for the case but also provides further research in legal CL with another tool to hold against the criticism of using potentially inappropriate corpora. Second, the combination of different approaches helps in making this case study more replicable and less susceptible to researcher bias. All in all, this case study is evidence of the applicability of corpus linguistic methods in questions of statutory interpretation.

## Footnotes

1 All examples are taken from COCA due to copyright reasons.
